# Effect of diurnal variation, *CYP2B6* genotype and age on the pharmacokinetics of nevirapine in African children

**DOI:** 10.1093/jac/dkw388

**Published:** 2016-10-05

**Authors:** Andrzej Bienczak, Adrian Cook, Lubbe Wiesner, Veronica Mulenga, Cissy Kityo, Addy Kekitiinwa, A. Sarah Walker, Andrew Owen, Diana M. Gibb, David Burger, Helen McIlleron, Paolo Denti

**Affiliations:** 1 Division of Clinical Pharmacology, Department of Medicine, University of Cape Town, Cape Town, South Africa; 2 MRC Clinical Trials Unit at University College London, London, UK; 3 Department of Paediatrics and Child Health, University Teaching Hospital, Lusaka, Zambia; 4 Joint Clinical Research Centre, Kampala, Uganda; 5 Baylor College of Medicine Bristol-Myers Squibb Children's Clinical Centre of Excellence, Kampala, Uganda/Gulu Regional Centre of Excellence, Gulu, Uganda; 6 Department of Molecular and Clinical Pharmacology, University of Liverpool, Liverpool, UK; 7 Department of Pharmacy, Radboud University Nijmegen Medical Centre, Nijmegen, The Netherlands

## Abstract

**Objectives:**

To characterize the effects of *CYP2B6* polymorphisms, diurnal variation and demographic factors on nevirapine pharmacokinetics in African children.

**Methods:**

Non-linear mixed-effects modelling conducted in NONMEM 7.3 described nevirapine plasma concentration–time data from 414 children aged 0.3–15 years.

**Results:**

Nevirapine pharmacokinetics was best described using a one-compartment disposition model with elimination through a well-stirred liver model accounting for a first-pass effect and transit-compartment absorption. Intrinsic clearance was affected by diurnal variation (characterized using a cosine function with peak amplitude 29% at 12 noon) and *CYP2B6* metabolizer status [extensive metabolizer (EM) 516GG|983TT, reference; intermediate metabolizer (IM) 516GT|983TT or 516GG|983TC, 17% lower; slow metabolizer (SM) 516TT|983TT or 516GT|983TC, 50% lower; ultra-slow metabolizer (USM) 516GG|983CC, 68% lower]. Age was found to affect pre-hepatic bioavailability: 31.7% lower at birth and increasing exponentially. Median (90% CI) evening *C*_min_ values in the different metabolizer groups were 5.01 (3.01–7.47), 6.55 (3.65–13.32), 11.59 (5.44–22.71) and 12.32 (12.32–27.25) mg/L, respectively. Evening *C*_min_ values were <3 mg/L in 43% of EM weighing <6 kg and 26% of IM weighing <6 kg, while 73% of SM and 88% of USM in all weight-bands had evening *C*_min_ values >8 mg/L. *C*_min_ was not markedly affected by administration time, but was altered by unequal splitting of the daily dose.

**Conclusions:**

Diurnal variation does not greatly affect nevirapine exposure. However, when daily doses cannot be split equally, the larger dose should be given in the morning. To achieve homogeneous exposures, nevirapine doses for SM and USM should be reduced by 50%, and children weighing <6 kg with EM or IM metabolizer status should receive the same dose as children weighing 6–10 kg.

## Introduction

Nevirapine was the first NNRTI available in low-income countries in a generic paediatric fixed-dose combination (FDC) tablet. This contributed to substantial cost reductions and improved the feasibility of treating HIV-infected children, and nevirapine is still widely used in resource-limited settings.^1–4^ Nevirapine has several advantageous characteristics: it has fewer drug interactions than PIs, it does not cause adverse CNS events when compared with efavirenz, and its bioavailability is not affected by food.^5^

Despite its high potency, nevirapine has a low genetic barrier for mutations and suboptimal drug exposures increase the risks of developing drug resistance and treatment failure.^6,7^ Several studies have reported highly variable nevirapine concentrations, with levels <3 mg/L among children in the lower paediatric weight-bands when dosed according to WHO guidelines, increasing the risk of virological failure.^1,2,8–12^ Nevirapine concentrations >8 mg/L, on the other hand, were associated with an increased risk of treatment discontinuation due to adverse events among adults.^7^ However, paediatric studies quantifying nevirapine pharmacokinetic variability due to different sources and suggesting optimization of current dosing remain limited.^8,13,14^

Nevirapine has a complex metabolism mediated mainly by *CYP3A4*- and *CYP2B6*-coded enzymes.^15^ SNPs present in *CYP2B6* (516G > T and 983T > C) were identified as the main source of nevirapine variability in adults,^16–18^ as for efavirenz.^4,18,19^ The prevalence of 516G > T loss of function (LOF) polymorphisms differs between populations and is particularly high in black Africans, whereas 983T > C variants are not observed among Caucasians.^4,18,19^ In our previous investigation of efavirenz pharmacokinetics in African children, we showed that extensive metabolizers (EM; *CYP2B6* 516GG|983TT genotype) are at higher risk of developing subtherapeutic efavirenz concentrations.^20^ A similar investigation of differences in nevirapine exposures between various metabolizer groups when dosed by weight-band according to current WHO guidelines has not yet been conducted in children. *CYP2B6* expression may be further modified by polymorphisms in genes coding nuclear receptors CAR (NR1|3) and PXR (NR1|2),^21,22^ although this has not been proved for nevirapine.^23^

The effect of the *CYP3A4* pathway on nevirapine pharmacokinetics is less studied. Although not confirmed for nevirapine, systemic exposures of *CYP3A* substrates have been shown to be altered by SNPs rs35599367 (CYP3A4*22)^24,25^ and rs776746 (CYP3A5*1).^26,27^ Additionally, *CYP3A* activity exhibits diurnal variation, with nevirapine clearance rates increasing during the day and reducing at night.^28,29^ Differences between morning (AM) and evening (PM) nevirapine trough concentrations (*C*_min_) have been previously reported^30^ and may relate to diurnal variation in the *CYP3A*-mediated effects on pharmacokinetics.

The aim of this analysis was: (i) to model the steady-state population pharmacokinetics of nevirapine in the largest cohort of African children studied so far; (ii) to quantify demographic and genotypic effects on nevirapine disposition; (iii) to characterize the effect of diurnal variation on nevirapine exposures under various dosing scenarios; and (iv) to propose optimal dosing strategies for this population.

Methods

In this analysis, sparsely sampled data from the CHAPAS-3 trial (Children with HIV in Africa—Pharmacokinetics and Adherence of Simple Antiretroviral Regimens)^31^ was enriched with intensive data from an earlier pharmacokinetic sub-study^1^ (part of CHAPAS-1).^32^ Both studies were conducted in African children from Uganda and Zambia, as briefly described below.

### CHAPAS-1

The trial evaluated dosing of, and adherence to, new paediatric FDC tablets: Triomune Baby (50 mg nevirapine, 6 mg stavudine and 30 mg lamivudine) and Junior (100 mg nevirapine, 12 mg stavudine and 60 mg lamivudine) in children <14 years dosed twice daily according to WHO 2006 guidelines.^33^ When the daily dose could not be split equally, the larger dose was given at night.

Children in the pharmacokinetic sub-study were sampled on one occasion at least 4 weeks after starting treatment. Samples were taken immediately prior to giving the morning dose and 1, 2, 4, 6, 8 and 12 h afterwards. The time of the preceding evening dose was assumed to be 12 h before the morning dose. Samples were stored and assayed using ultra HPLC with UV detection at the Department of Pharmacy of the Radboud University Medical Centre, Nijmegen, The Netherlands. The method was linear over the range of 0.1–10 mg/L. The average intra-assay and inter-assay coefficients of variation (CV) and relative error (RE) were 2.9%, 2.4% and 97%, respectively.^34^

### CHAPAS-3

Pharmacokinetics, toxicity, acceptability, adherence and virological efficacy were compared between three first-line antiretroviral regimens in children 13 years or younger.^31^ Depending on treatment allocation, patients received: Triomune Baby, Triomune Junior, Duovir-N Baby (50 mg nevirapine, 60 mg zidovudine and 30 mg lamivudine) or nevirapine (100 mg)—all paediatric formulations; or Duovir-N (200 mg nevirapine, 300 mg zidovudine and 200 mg lamivudine) or Triomune30 (200 mg nevirapine, 30 mg stavudine and 150 mg lamivudine), formulated for adults. Nevirapine-based regimens were dosed twice daily according to WHO 2010 guidelines.^35^ When the daily dose could not be split equally, the larger dose was given in the morning.

Children on nevirapine were sampled during clinic visits at week 6, week 36 and every 24 weeks thereafter until the end of the study; at each visit two samples were taken at least 2 h apart. The self-reported times of the morning and penultimate doses were recorded. Samples were stored and analysed by LC-tandem MS at the Division of Clinical Pharmacology, University of Cape Town, South Africa. The method was linear over the range of 0.0195–20 mg/L. The average intra-assay and inter-assay CV and RE were 2.9%, 2.4% and 97%, respectively.

### Genotyping

Genotyping was performed only on patients from CHAPAS-3 by allelic discrimination real-time PCR assay on a DNA Engine Chromo4 system (Bio-Rad Laboratories, Inc., Hercules, CA, USA). The PCR protocol involved an initial denaturation step at 95°C for 15 min, followed by 50 cycles of amplification at 95°C for 15 s and final annealing at 60°C for 1 min. TaqMan^®^ Genotyping Master Mix and assays for *CYP2B6* 516G > T (rs3745274; ID: C_7817765_60), *CYP2B6* 983T > C (rs28399499; ID: C_60732328_20), *CYP2B6* 15582C > T (rs4803419; ID: C_7817764_10), CYP3A4*22 (rs35599367, C__59013445_10), *CYP3A5* 6986G > A (rs776746, C__59013445_10), *NR1I3* (rs3003596, C__16194070_10 and rs2307424, C__25746794_20), *NR1I2* 63396C > T (rs2472677, C__26079845_10), and *ABCC10* (rs2125739, C__16173668_10) were obtained from Life Technologies Ltd (Paisley, UK). Opticon Monitor^®^ version 3.1 (Bio-Rad Laboratories) was used to obtain allelic discrimination plots and make allele calls.

The distribution of the genotypes was tested for Hardy–Weinberg equilibrium using the exact test in the R ‘genetics’ package.

### Population pharmacokinetic analysis

#### Model building

The steady-state pharmacokinetics of nevirapine was analysed using non-linear mixed-effects modelling with NONMEM 7.3^36^ and the first-order conditional estimation method with interaction. PsN 4.4.0, Pirana and Xpose were used to facilitate modelling and for model diagnostics.^37^ Model building was conducted starting with intensive pharmacokinetic data from CHAPAS-1 followed by sparse data from CHAPAS-3.^38^ The stepwise process was guided by differences in the NONMEM objective function value (OFV; proportional to −2 log-likelihood), inspection of goodness-of-fit (GOF) plots and visual predictive checks (VPCs), biological plausibility and clinical relevance. OFV drops >3.84 between two hierarchical models after adding one parameter were considered a significant improvement (*P *≤ 0.05, χ^2^-distribution, df = 1). The stability and robustness of the final model, together with the precision of parameter estimates, was evaluated using non-parametric bootstrap (*n *= 50, due to long model run times).

The model-derived empirical Bayesian estimates for the individual parameters were used to predict morning and evening *C*_min_ and AUC_0–12_ (area under the concentration–time curve between dosing events) at steady state for each sampling occasion and patient.

#### Structural model

One-, two- and three-compartment disposition models with first-order absorption and elimination were tested, as well as delayed and transit-compartment^39^ absorption. A semi-mechanistic well-stirred hepatic extraction model was tested for elimination, as in Gordi *et al*.^40^ This hepatic model assumed the following parameters: nevirapine fraction unbound in plasma (*f_u_*) 40%,^41^ hepatic plasma flow (Q_H_) 50 L/h^42^ and liver volume (V_H_) 1 L^40^ for a typical 70 kg individual (allometrically scaled).

Between-subject variability (BSV) and between-occasion variability (BOV) were tested on all pharmacokinetic parameters assuming log-normal distribution. Residual unexplained variability (RUV) was described using a combined proportional and additive structure. We excluded from the analysis data with uncertain dosage history and nevirapine concentrations below the limit of quantification (BLQ), presumed to be due to non-compliance^38^ (confirmed by undetectable concentrations of the companion antiretroviral drugs). Further implausible outliers were identified using visual checks and excluded based on conditional weighted residuals (|CWRESI| > 3).

#### Covariate effects

Allometric scaling was added to the model at an early stage (before covariate testing), as suggested by Anderson and Holford,^43^ and applied to all clearance and volume parameters. For intrinsic clearance (CL_int_) and pre-hepatic bioavailability (F_preH_) we tested the effect of age using a power, hockey-stick, exponential or sigmoidal function with/without Hill coefficient models.^43^ The effect of diurnal variations was investigated using step or cosine functions.^29^ Besides weight and age, the other covariates tested were: study site, NRTI treatment backbone, sex, weight-for-age Z-score (WAZ), height-for-age Z-score (HAZ) and formulation. Pharmacogenetic effects were tested as individual SNPs (rs3745274, rs28399499, rs4803419, rs35599367, rs776746, rs3003596, rs2307424, rs2472677, rs2125739) and as metabolizer status determined by SNPs 516G > T and 983T > C [EM, genotype 516GG|983TT; intermediate metabolizer (IM), single variant allele (516GT|983TT or 516GG|983TC); slow metabolizer (SM), two variant alleles (516TT|983CC or 516GT|983TC); ultra-slow metabolizer (USM), 983CC irrespective of 516G > T genotype].

Mixture modelling with frequencies fixed to those observed in the study population was used to impute missing genotypes (predominantly in CHAPAS-1).^44^ Proportionality and correction factors were applied on RUV to test for differences between the assays and laboratories used.

#### Simulations

For the simulation (conducted with NONMEM 7.3), the demographics of the 414 patients (weight 3.5–29.6 kg) from the original analysis were used and enriched with 116 records of individuals weighing 20–35 kg from CDC Growth Charts (age and corresponding median weight used).^45^ The final model was used to simulate exposures after nevirapine administration under various dosing scenarios and assuming 3–8 mg/L as the therapeutic range for nevirapine.^46^ Each *in silico* patient was resimulated 100 times, changing their metabolizer status according to the proportions in the study population, which ensured the same distribution in each weight-band. The effect of drug intake time (6:00, 7:00, 8:00, 9:00 AM/PM) and dose-splitting strategies (AM/PM D1:100/50 mg, D2:75/75 mg, D3:50/100 mg) was studied in a single patient (0.44 years, 7.2 kg, IM) simulated 1000 times. To avoid generating implausibly extreme values, the maximum variability for each random effect was limited to 3 standard deviations. Data analysis and plot generation was performed using R.^47^

## Results

### Demographic characteristics and samples

This analysis included 3305 samples (539 in intensive and 2766 in sparse pharmacokinetic profiles) from 414 African children (78 CHAPAS-1, 330 CHAPAS-3, 6 in both). Baseline demographic characteristics are presented in Table 1; 246 samples were excluded from the analysis (111 due to unclear dosage history, 87 outliers and 48 BLQ). Genotypes were available for 324 children (Table S1, available as Supplementary data at *JAC* Online); *CYP2B6* metabolizer groups were 33.1% EM, 44.6% IM, 21.7% SM and 0.6% USM (Table 2); the mixture-model allocation for the remaining 96 individuals was 41.7% EM, 49.0% IM and 9.4% SM. All tested genotypes were in Hardy–Weinberg equilibrium (Table S1).

**Table 1.** Demographic characteristics

Characteristic	CHAPAS-1	CHAPAS-3	Combined
No. of children	84	336	414
No. of samples included	539	2766	3305
No. of samples excluded (BLQ)	8 (0)	238 (48)	246^a^
No. of sampling occasions, *n* or median (range)	1	3 (1–7)	3 (1–8)
Age (years)^b^, median (range)	6.2 (0.4–15.0)	2.6 (0.3–12.2)	2.92 (0.3–15.0)
Weight (kg)^b^, median (range)	15.75 (3.5–29.0)	11.5 (4.9–29.6)	12.2 (3.5–29.6)
WAZ, median (range)	−1.1 (−4.2–2.0)	−1.7 (−7.2–1.2)	−1.5 (−7.2–2.0)
Male/female, *n*/*n*	52/32	177/159	80/89
NRTI, *n*			
abacavir	0	115	115
stavudine	84	107	191
zidovudine	0	114	114

Six patients rolled over from CHAPAS-1 to CHAPAS-3; all patients were black Africans.

^a^Samples excluded from the analysis: unclear dosage history, 111; implausible (visual check confirmed by |CWRES| > 3), 87; and BLQ confirmed by undetectable levels of the companion drugs, 48.

^b^Baseline values.

**Table 2.** Exposures of different metabolic subgroups determined by 516G>T|983T>C SNP vector

Metabolizer status	Patients, *n* (%)	*C*_minAM_ (mg/L), median (5th–95th percentile)	*C*_minPM_ (mg/L), median (5th–95th percentile)	*C*_minPM_ <3 mg/L^a^, *n* (%)	*C*_minPM_ 3–8 mg/L^a^, *n* (%)	*C*_minPM_ >8 mg/L^a^, *n* (%)	AUC_AM_ (mg · h/L), median (5th–95th percentile)	AUC_PM_ (mg · h/L), median (5th–95th percentile)
EM	106 (33.3)	5.01 (3.01–7.47)	4.58 (2.53–7.03)	77 (16.6)	361 (77.6)	27 (5.8)	68.51 (39.42–104.16)	69.34 (38.65–104.42)
IM	141 (44.2)	6.55 (3.65–13.32)	6.08 (3.25–12.93)	33 (5.8)	378 (66.8)	155 (27.4)	88.93 (50.06–173.72)	88.60 (50.06–173.72)
SM	70 (21.9)	11.59 (5.44–22.71)	10.91 (5.06–22.44)	4 (1.3)	78 (25.7)	222 (73.0)	152.07 (72.42–270.46)	151.27 (71.54–287.46)
USM	2 (0.6)	12.32 (12.32–27.25)	11.71 (11.71–26.43)	0 (0)	1 (12.5)	7 (87.5)	170.81 (170.81–362.97)	152.12 (152.12–337.26)

EM, 516GG|983TT; IM, 516GG|983TC or 516GT|983TT; SM, 516TT|983TT or 516GT|983TC; USM, 516GG|983CC.

Data for 319 individuals from the CHAPAS-3 trial with available genotype dosed according to WHO 2010 guidelines^35^ corresponding to 1343 pharmacokinetic visits. When multiple pharmacokinetic visits were available, measurements were used to calculate the geometric mean for every patient, which were then used to calculate median and percentiles in each subgroup.

^a^Number of *C*_min_ below, within and above the therapeutic range of 3–8 mg/L.^45^

### Population pharmacokinetics

**Figure 1.**
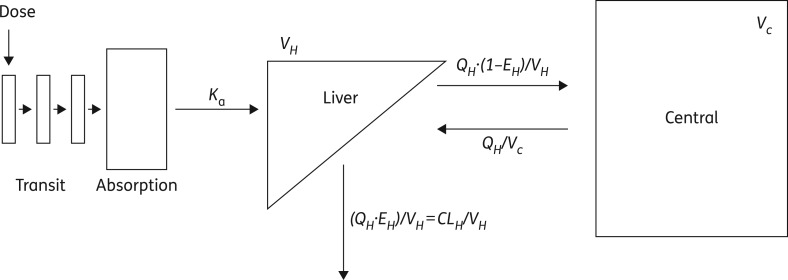
Compartmental structure of the nevirapine pharmacokinetic model. CL_H_, hepatic clearance; E_H_, hepatic extraction; *K*_a_, absorption rate constant; Q_H_, hepatic plasma flow; V_H_, volume of the liver; V_C_, volume of the central compartment. The model parameters and presented relations are explained in detail in Appendix S1.

Nevirapine pharmacokinetics were best described using one-compartment disposition, absorption through transit compartments and elimination using the semi-physiological model with first-pass hepatic extraction (Figure 1 and Appendix S1). The final model parameters were estimated relative to pre-hepatic bioavailability (F_preH_, with typical value fixed to 1) and are presented in Table 3. All parameter estimates were found to be reasonably robust and adequate model fit was confirmed through GOF and VPC plots, which showed adequate fit of our model to the analysed data (Figures S1 and S2).

**Table 3.** Final parameter estimates (5th–95th percentile)^a^

Parameter	Typical values	Variability (%)^b^
CL_int_		
EM (L/h)	3.27 (3.00–3.69)	BSV CL_int_: 21.40 (20.08–32.46)
IM (L/h)	2.72 (2.27–2.94)	
SM (L/h)	1.65 (1.47–1.89)	
USM (L/h)	1.04 (0.87–1.38)	
AMP (%)	29.2 (27.7–45.2)	
SHIFT (h)	−12.30 (−13.32 to −10.38)	
V_C_ (L)	21.92 (20.24–26.23)	
F_preH_		
older children^c^	1 (fixed)	BSV F_preH_: 18.72 (6.59–20.66)
at birth (%)	58.30 (50.48–68.24)	BOV F_preH_: 17.02 (16.12–20.87)
*t*_1/2_ (years)	1.54 (1.47–2.58)	
Increased BOV F_preH_ for unobserved intake	1.54 (1.20–1.65)	
MTT (h)	0.56 (0.49–0.70)	BOV MTT: 199.73 (177.23–217.70)
*K*_a_ (1/h)	0.84 (0.67–1.12)	BOV *K*_a_: 44.91 (31.32–50.46)
N_TRANS_ (number)	3 (fixed)	
Additive error (mg/L)	0.32 (0.21–0.38)	
Proportional error (%)	5.26 (4.26–6.18)	
Increased error for sparse data	1.56 (1.49–1.81)	

CL_int_, intrinsic clearance; AMP, amplitude of cosine function; SHIFT, shift in the zenith of cosine function from midnight; V_C_, volume of central compartment; F_preH_, pre-hepatic bioavailability; N_TRANS_, number of transit compartments (in the implementation of Savic *et al*.^39^ this would be NN = 2); MTT, absorption mean transit time; *K*_a_, absorption rate constant; BSV, between-subject variability; BOV, between-occasion variability.

Final parameter estimates are typical population values estimated by the model. All clearance and volume parameters scaled allometrically to the median weight of 14.5 kg.

The number of transit compartments was first estimated and then fixed during the covariate analysis in order to improve model stability. The number was then re-estimated in the final model and proved not to be different from that previously fixed. The equations explaining the relation between presented parameters can be found in Appendix S1.

^a^Estimated from non-parametric bootstrap (*n *= 50) of the final model.

^b^Expressed as approximate %CV on SD scale (√ETA×100).

^c^Older children refers to individuals where no further age-driven increase in bioavailability can be observed (Figure 3).

**Figure 2.**
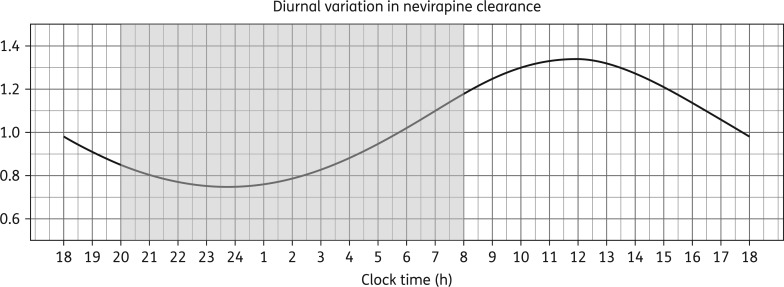
Diurnal variation in nevirapine intrinsic clearance detected by the model, presented over 24 h. The shaded area corresponds to night-time (20.00–08.00 h).

Implementing the well-stirred liver model decreased OFV by 42, without adding extra parameters. The model was parameterized with CL_int_ following a circadian rhythm expressed through oscillations of the cosine function with zenith around 12 noon and amplitude of ∼29% (ΔOFV = −91, df = 2, *P *< 0.001) (Figure 2). The model identified distinct pre-hepatic (F_preH_) and hepatic components (F_H_) of bioavailability, since changes in liver activity mechanistically affected also F_H_. The reference value of F_preH_ was fixed to 1, and BSV and BOV were estimated. Including the diurnal effect reduced BSV in CL_int_ by 34% and BOV in F_preH_ by 41%. More details on the model implementation, including formulae explaining the relationship between model parameters, are presented in Appendix S1.

After applying allometric scaling to account for the effect of body size, and including diurnal effects and first-pass metabolism, the most significant covariate was the metabolizer status on CL_int_ determined by *CYP2B6 *516G > T|983T > C genotype (ΔOFV = −217, df = 3, *P *< 0.001), explaining 85% of remaining BSV in CL_int_. Using six rather than four 516G > T|983T > C SNP-vector metabolizer groups^20^ reduced OFV by only 5 points (df = 2, *P *= 0.08) and was therefore not used.

**Figure 3.**
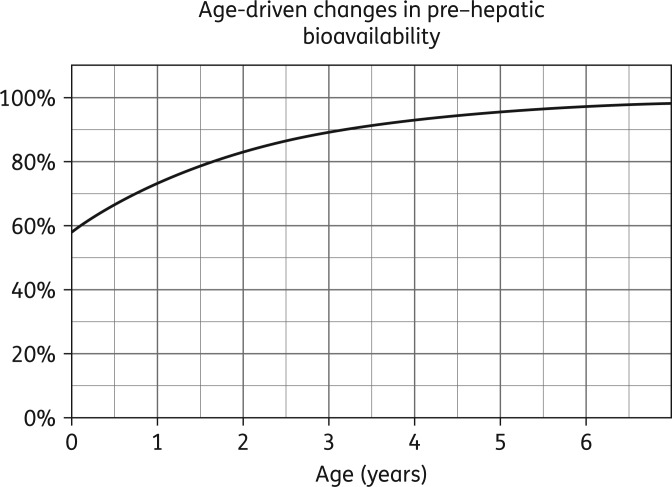
Change in nevirapine pre-hepatic bioavailability with age.

Our data did not support a maturation effect on CL_int_, but we identified age-driven differences in F_preH_, which were described using an exponential model [Equation (7) in Appendix S1]. F_preH_ at birth was estimated as 58.3% of the value in older children (reference fixed to 100%), 90% of F_preH_ was reached by age of ∼3.3 years and the half-life of the process was 1.55 years (Figure 3).

The model estimated that an average child weighing 14.5 kg and aged 4.1 years would have F_preH _= 93% and their values of oral clearance (CL_oral_, see Appendix S1 and Table S2) were 1.31 L/h EM (reference), 1.09 L/h IM (17% lower), 0.66 L/h SM (50% lower) and 0.42 L/h USM (68% lower). A summary of the individual exposures in children from the CHAPAS-3 trial dosed according to WHO 2010 guidelines^35^ is presented in Table 2, split by metabolizer genotype.

Higher uncertainty related to unobserved intake time (for all sparse data and pre-dose samples in intensive data) was accounted for by scaling factors (proportional model) on RUV and BOV F_preH_, which were found to be respectively 1.56 and 1.54 times larger than in pharmacokinetic samples after observed intake.

No other covariates were identified as significant. The remaining stochastic variability in clearance and bioavailability was low (BSV CL_int_ 21.4%, BSV F_preH_ 18.7% and BOV F_preH_ 17%), but absorption parameters (where no covariates improved model fit) remained highly variable [BOV absorption rate constant (*K*_a_) 44.9%, BOV absorption mean transit time (MTT) 199.7%].

### Simulations

**Figure 4.**
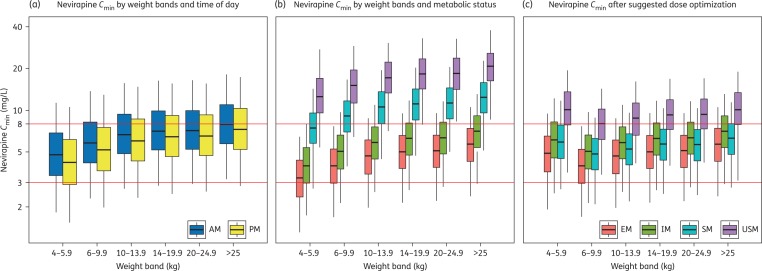
Model-simulated exposures shown by dosing weight-bands. (a) Difference between morning and evening *C*_min_ when dosed according to current WHO recommendations.^35^ (b) Difference in *C*_min_ between metabolic groups when dosed according to current WHO recommendations (evening *C*_min_ is shown). (c) Difference in *C*_min_ between metabolic groups when dosed according to the proposed dose optimization strategy (evening *C*_min_ is shown). Red horizontal lines correspond to the nevirapine therapeutic range, from 3 to 8 mg/L. The boxes in the percentile plots show the 25th percentiles, medians and 75th percentiles, while the whiskers correspond to the 5th and 95th percentiles of the simulated data. EM, 516GG|983TT; IM, 516GG|983TC or 516GT|983TT; SM, 516TT|983TT or 516GT|983TC; USM, 516GG|983CC. This figure appears in colour in the online version of *JAC* and in black and white in the print version of *JAC*.

Simulations were conducted to compare average *C*_minAM_ and *C*_minPM_ in weight-bands of African children divided into metabolizer groups and dosed following WHO 2010 recommendations.^35^ Average *C*_minAM_ and *C*_minPM_ in weight-bands >6 kg were >3 mg/L for most simulated individuals regardless of metabolizer status (Figure 4a). In contrast, >25% of children in the lowest weight-band (4–6 kg) had *C*_minPM_ below the efficacy threshold (Figure 4b); this effect was driven mostly by EM and IM (43% and 26% <3 mg/L, respectively).

Given the detected diurnal variation in nevirapine CL_int_, we evaluated the effect of administration time (see the Methods section) on average morning and evening exposures. The changes in median concentration depending on administration time and differences in systemic drug exposures are presented in Figure S3. Depending on administration time, the ratios of morning/evening exposures varied between 1.09–1.15 for *C*_min_ and 1.03–1.07 for AUC_0–12_, differences that are unlikely to be clinically significant.

Use of some nevirapine FDCs can lead to unequal splitting of the advised daily dose between morning and evening intakes. Simulation results showed that ratios between simulated median *C*_minAM/PM_ for tested dose-splitting strategies (see the Methods section) were: D1 (larger morning) 0.93, D2 (equal) 1.13 and D3 (larger evening) 1.41; and AUC_0–12_ 0.90, 1.04 and 1.22, respectively (Figure S4).

## Discussion

We present the largest investigation to date of nevirapine pharmacogenetics, the first report of the effect of 983CC homozygosity on nevirapine pharmacokinetics and the first study in children to quantify the combined effect of *CYP2B6* 516G > T|983T > C. Our analysis is also the first to date to characterize the diurnal variation in nevirapine clearance through population pharmacokinetic modelling and to evaluate the effect of this phenomenon on systemic drug exposures through simulations.

The main predictor of nevirapine clearance in our cohort of African children was the combined effect of the *CYP2B6* 516G > T|983T > C genotype. Oral clearance estimated by our model before adjusting for the *CYP2B6*-SNPs was 3.8 L/h, comparable to the 3.93 L/h reported previously in children^13^ (both scaled up to 70 kg) and the 2.82–3.97 L/h found in adults.^16,17,38,48–53^ Comparing the *CYP2B6* 516G > T|983T > C effect with other reports is problematic, since our study is the first to use this categorization with four metabolizer subgroups for nevirapine, although it has been extensively applied to efavirenz.^20,54^ The 50% lower nevirapine clearance we detected for SM is greater than the 30%–37% drop previously reported for 516TT versus 516GG.^8,14,16,17,55,56^ Similar to efavirenz,^18,20^ the effect of *CYP2B6* 983CC (recessive homozygosity) on nevirapine pharmacokinetics is of greater magnitude than that of 516TT (68% versus 50% drop). Unsurprisingly, nevirapine clearance is affected to a lesser degree by *CYP2B6* polymorphisms than efavirenz in the same population.^20^ This can be explained by a different contribution of the *CYP3A4* pathway to the metabolism of these drugs.^57^

Diurnal variation has been previously documented for several *CYP3A4* substrates,^58,59^ consistently revealing increased clearance rates during the day as compared with during the night.^28,60,61^ Our study replicated those findings and detected significantly higher nevirapine clearance during the day, with a maximum at midday. The estimated amplitude of the diurnal variation is somewhat larger than previous reports in *CYP3A4* probes.^28,60^ This could be due to the considerable contribution of *CYP2B6* enzymes to nevirapine clearance. Although little is known about the chrono-pharmacokinetics of this pathway, our hypothesis is supported by the fact that CAR, which regulates expression of *CYP2B6*,^21^ exhibits a circadian rhythm linked to a 1.7-fold magnitude induction of *CYP2B* mRNA.^62^

Despite the 29% amplitude of diurnal variation in nevirapine clearance, the simulated difference between morning and evening trough exposures was <15%. This lack of effect is due to nevirapine's relatively long half-life (25–30 h at steady-state)^41^ in comparison with, for example, PIs, where the reported median difference in troughs is almost 60%.^61^ Simulations revealed only a marginal effect of intake time on exposures, but showed that the diurnal variation should be considered when the daily dose of nevirapine cannot be split equally, since 50% difference in the ratio of median *C*_min_ AM/PM was found depending on whether the larger dose is given in the morning or evening. To minimize this effect and the risk of suboptimal exposures, uneven splitting should be implemented with the larger dose given in the morning, which is currently not specified in the WHO guidelines.^35^

A further innovation of our study was the use of a semi-physiological well-stirred liver model, allowing the effect of hepatic clearance (expressed as intrinsic clearance) on both systemic clearance and first-pass hepatic extraction to be captured, so that clearance and its covariates affect bioavailability. This model allowed us to separate the pre-hepatic and hepatic components of bioavailability.

A significant degree of variability in nevirapine pharmacokinetics was explained in our model by age-driven differences in pre-hepatic bioavailability, which possibly overshadowed the expected effect of maturation of the metabolic pathways. A similar effect was found for nevirapine by Foissac *et al.*^13^ and reported for other antiretroviral drugs^63^ and could hypothetically be caused by reduced drug absorption in neonates and younger children. This may be due to more rapid gastric emptying, smaller gastric volume, higher gastric pH, smaller gastrointestinal absorption area, as well as adherence and palatability issues.^64^ This phenomenon could explain the subtherapeutic concentrations seen in the youngest age groups in other paediatric studies.^1,2,8–12^ Our simulations show in particular that individuals in the <6 kg weight-band who are EM and IM are at risk of suboptimal exposures (observed evening *C*_min_ <3 mg/L in 43% and 26% of individuals, respectively).

Despite significant differences in nevirapine pharmacokinetics determined by *CYP2B6* genotype, a genotype-driven dose optimization strategy has not been previously suggested. This could be due to the fact that, unlike efavirenz, the relationship between high exposures and toxicity is not strongly apparent.^7,16,65^ Nonetheless, suboptimal concentrations are of concern, as they could lead to virological failure.^7–9,13^ To prevent suboptimal exposures we suggest the dose for EM and IM in the lowest weight-band be increased from 100 to 150 mg. Further harmonization of exposures across metabolizer groups could be achieved by 50% reduction of nevirapine dose for SM and USM in all other weight-bands, as >75% of those children had evening *C*_min_ above the 8 mg/L therapeutic upper limit, although this might be of limited clinical relevance. When the daily dose cannot be split equally, larger doses should be given in the morning. The simulated *C*_min_ based on this dose-optimization approach are presented in Figure 4(c). We acknowledge, however, that practical implementation of such a strategy in resource-limited settings would be hindered by restricted access to genotyping and current use of FDCs.

Our study has several limitations. The therapeutic range for nevirapine used in our analysis has not been previously evaluated in children or in African populations. The intake time for the sparse pharmacokinetic data was self-reported and might be inaccurate, given the large variability in absorption parameters, and could inflate the magnitude of the detected diurnal variation. We tried to minimize this effect by excluding samples with uncertain dosage information and BLQ. The detected diurnal effect could hypothetically be further affected by food intake, which was not recorded in our study. However, food has been previously reported not to modify nevirapine bioavailability or clearance.^5^ Additionally, the analysed trials differed in the morning/evening dose-splitting strategy (see the Methods section), but the model-based approach we employed accounts for this difference.

### Conclusions

This is the first study quantifying the combined effect of *CYP2B6* 516G>T|983T>C on nevirapine clearance in children and classifying metabolizers into four metabolic groups (EM, IM, SM and USM). To prevent subtherapeutic exposures, EM and IM children weighing <6 kg should receive same the dose as those in the 6–10 kg weight-band. Further homogenization of exposures can be achieved by reducing the current recommended dose for SM and USM by 50% in other weight-bands. Additionally, we characterized the effect of diurnal variation on nevirapine pharmacokinetics, and found that it is of limited clinical relevance, possibly due to nevirapine's long half-life. However, this phenomenon should be taken into consideration when daily doses cannot be split equally and larger doses should be given in the morning.

## Funding

CHAPAS-1 was supported by the European and Developing Countries Clinical Trials Partnership (EDCTP, 2004.01.H.d2.33011). Cipla Ltd donated first-line antiretrovirals.

CHAPAS-3 was supported by EDCTP (IP.2007.33011.006), Medical Research Council UK, Department for International Development UK and Ministerio de Sanidad y Consumo Spain. Cipla Ltd donated first-line antiretrovirals.

The drug assays were supported in part by the National Institute of Allergy and Infectious Diseases of the National Institutes of Health (UM1 AI068634, UM1 AI068636 and UM1 AI106701, U01 AI068632), the Eunice Kennedy Shriver National Institute of Child Health and Human Development (NICHD) and the National Institute of Mental Health (AI068632).

## Transparency declarations

A. B., A. C., V. M., C. K., A. K., A. S. W., D. M. G., D. B. and H. M. have received support through grants from the European Developing Countries Clinical Trials Partnership (EDCTP). A. C., A. K., A. S. W. and D. M. G. have additionally received grants from the Medical Research Council (MRC) UK. H. M. additionally declares support in part by the National Research Foundation of South Africa, grant 90729. A. O. has received support in form of grants from Janssen, ViiV and Tandem Nano, as well as personal fees from Merck and was issued a patent ‘Compositions of efavirenz’. L. W. and P. D.: none to declare.

## Disclaimer

The content is solely the responsibility of the authors and does not necessarily represent the official views of any funders.

## Supplementary data

Tables S1 and S2, Appendix S1 and Figures S1–S4 are available as Supplementary data at *JAC* Online (http://jac.oxfordjournals.org/).

Supplementary Data
