# Supplementary material for: Effect of diurnal variation, CYP2B6 genotype and age on the pharmacokinetics of nevirapine in African children
Source: J Antimicrob Chemother. 2016 Oct 5;72(1):190–9. doi: 10.1093/jac/dkw388 (PMC5161049; doi:10.1093/jac/dkw388)
Supplement: Supplementary Data [file supp_72_1_190__index.html]

Effect of diurnal variation, CYP2B6 genotype and age on the pharmacokinetics of nevirapine in African children — Effect of diurnal variation, CYP2B6 genotype and age on the pharmacokinetics of nevirapine in African children — Supplementary Data 

# Effect of diurnal variation, *CYP2B6* genotype and age on the pharmacokinetics of nevirapine in African children

## Supplementary Data

Supplementary Data

- Supplementary Data - Doc file
