# Supplementary material for: Effect of diurnal variation, CYP2B6 genotype and age on the pharmacokinetics of nevirapine in African children
Source: J Antimicrob Chemother. 2016 Oct 5;72(1):190–9. doi: 10.1093/jac/dkw388 (PMC5161049; doi:10.1093/jac/dkw388)
Supplement: Supplementary Data [file supp_dkw388_dkw388supp.doc]

**Supplementary data**

**Appendix S1**

The nevirapine model presented in Figure 1 is explained in detail below.

The delay between oral administration and absorption is modelled through 2 transit compartments. After entering the absorption compartment, nevirapine is transferred to the liver, where it undergoes 1^st^-pass hepatic extraction (E_H_). The fraction of the drug not eliminated by 1^st^-pass (1-E_H_) is then transported via hepatic plasma flow (Q_H_) to the central compartment and the systemic circulation. It then recirculates back to the liver, which is the site of drug clearance. In this well-stirred model, hepatic clearance (CL_H_) is determined by Q_H_ and E_H_ as follows:

(1)
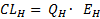


E_H_ depends on the unbound fraction of the drug (f_u_), liver activity (CL_int_), and Q_H_ and is defined as:

(2)
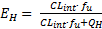


E_H_ also determines the hepatic bioavailability F_H_

(3)
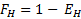


The total oral bioavailability (F) is determined by both the pre-hepatic (F_preH_) and hepatic (F_H_) components, as follows:

(4)
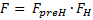


After a number of transformations, oral clearance can be simplified as follows:

(5)
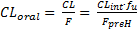


Due to circadian rhythm variations, CL_int_ changes with time, thus affecting both CL_H_ and F_H_, and its value at time (t) is defined as follows:

(6)
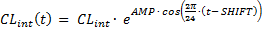


where AMP is the amplitude of the cosine oscillation and SHIFT is the phase shift of the cosine function relative to 00:00. In order to prevent negative values of CL_int_ the effect of the circadian rhythm was modelled as exponential and can be interpreted approximately as a relative change.

Furthermore, F_preH_ changes with age, as expressed by following equation:

(7)
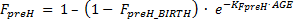


where FpreH_BIRTH is the F_preH_ at birth, K_FpreH_ is the rate constant for age-driven change in F_preH_ and AGE refers to age.

| **Table S1. Observed Frequencies of Tested Single Nucleotide Polymorphisms with Corresponding Hardy-Weinberg P-values** | | | | | | |
| --- | --- | --- | --- | --- | --- | --- |
| **Gene** | **SNP** | **Hom-Ref†** | **Het-LOF†** | **Hom-LOF†** | **MAF** | **HWE**  **P-value** |
| ***CYP2B6*** | **rs3745274 (516G>T)** | GG | GT | TT | 0.36 | 0.18 |
|  |  | 136 (0.43) | 136 (0.43) | 47 (0.15) |  |  |
|  | **rs28399499 (983T>C)** | TT | TC | CC | 0.09 | 1 |
|  |  | 226 (0.83) | 51 (0.16) | 2 (0.01) |  |  |
|  | **rs4803419 (15582C>T)** | CC | TC | TT | 0.07 | 0.19 |
|  |  | 227 (0.87) | 39 (0.12) | 3 (0.01) |  |  |
| ***CYP3A4*** | **rs35599367 (CYP3A4*22)** | GG | GA | AA | 0.003 | 1 |
|  |  | 317 (0.99) | 2 (0.01) | 0 |  |  |
| ***CYP3A5*** | **rs776746 (6986G>A)** | GG | GA | AA | 0.82 | 0.44 |
|  |  | 12 (0.04) | 88 (0.28) | 219 (0.69) |  |  |
| ***NR1I3 (CAR)*** | **rs3003596** | AA | AG | GG | 0.49 | 1 |
|  |  | 78 (0.24) | 159 (0.50) | 82 (0.26) |  |  |
|  | **rs2307424**  **(540C>T)** | CC | CT | TT | 0.08 | 0.42 |
|  |  | 272 (0.85) | 44 (0.14) | 3 (0.01) |  |  |
| ***NR1I2 (PXR)*** | **rs2472677 (63396C>T)** | CC | CT | TT | 0.36 | 0.14 |
|  |  | 124 (0.39) | 160 (0.50) | 35 (0.11) |  |  |
| ***ABCC10*** | **rs2125739** | TT | CT | CC | 0.23 | 0.27 |
|  |  | 185 (0.58) | 120 (0.38) | 13 (0.04) |  |  |

**†**number (proportion). Hom-Ref - homozygous for the functional allele; Het-LOF - heterozygous for the loss-of-function (LOF) allele; Hom-LOF - homozygous for the LOF allele; MAF – minor allele frequency; HWE - Hardy-Weinberg equilibrium.

Note: information for 319 children from CHAPAS-3 study (aside from rs2125739 – data on 318 children).

| **Table S2. Model estimated clearance intrinsic and corresponding hepatic clearance, hepatic extraction, hepatic bioavailability and oral clearance by metaboliser status.** | | | | | |
| --- | --- | --- | --- | --- | --- |
| **Metabolizer Status** | **CL_int_ [L/h]** | **CL_H_ [L/h]** | **E_H_** | **F_H_** | **CL_oral_**  **[L/h]** |
| **Fast** | 3.27 | 1.20 | 7.9% | 91.1% | 1.29 |
| **Intermediate** | 2.72 | 1.01 | 6.6% | 93.4% | 1.09 |
| **Slow** | 1.65 | 0.63 | 4.1% | 95.9% | 0.68 |
| **Very slow** | 1.04 | 0.40 | 2.6% | 96.4% | 0.43 |

CL_int_ – clearance intrinsic; CL_H_ – clearance hepatic; E_H_ – hepatic extraction; F_H_ – hepatic component of bioavailability; CL_oral_ – oral clearance

Note: The relationship between parameters and how they can be derived explained in the Appendix. Presented values relate to an average child of 14.5 kg, 4.1 years of age and corresponding pre-hepatic bioavailability of 93% and hepatic plasma flow of 15.35 (L/h).


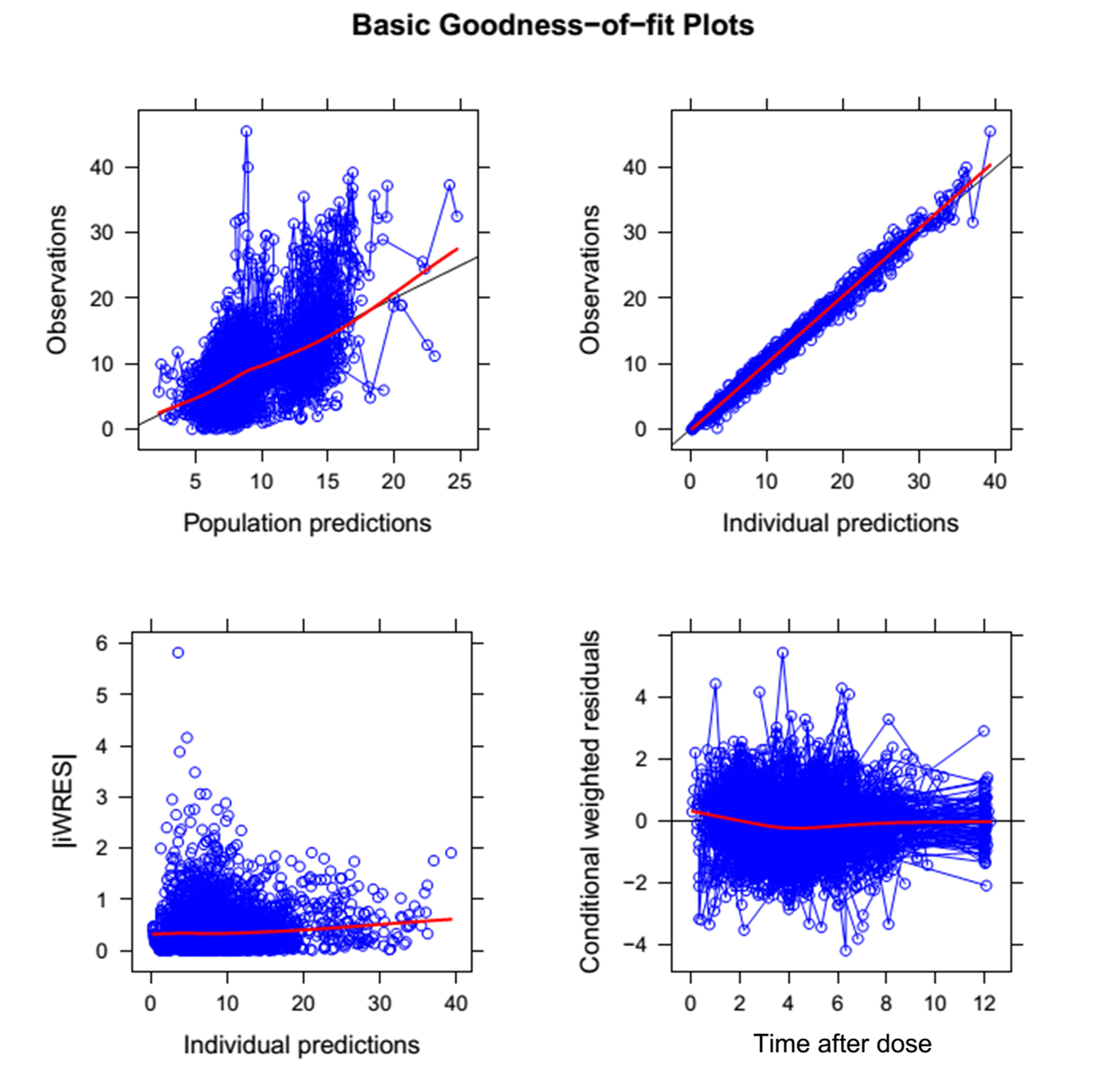


**Figure S1.** Goodness of fit plots. Top left – observations vs population predictions (log scale); top right – observations vs individual predictions (log scale); bottom left – absolute values of individual weighted residuals vs individual predictions bottom right – conditional weighted residuals (CWRESI) vs time after dose;


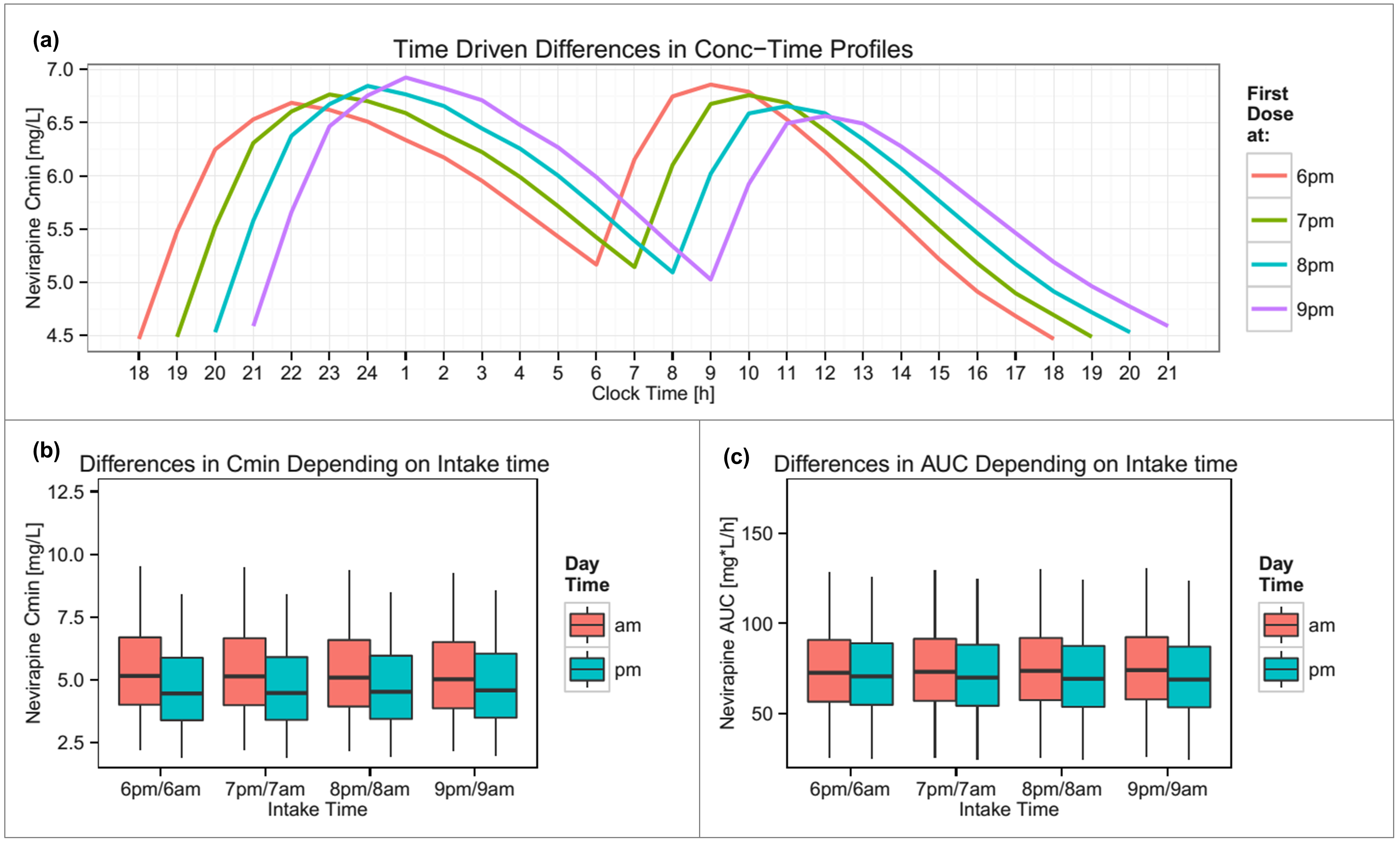


**Figure S2.** Results of simulations evaluating the effect of intake time on nevirapine exposures (see Methods): (a) concentration-time curves for evaluated intake time scenarios; (b) differences between morning and evening C_min_ depending on intake time; (c) differences between morning and evening AUC depending on intake time.


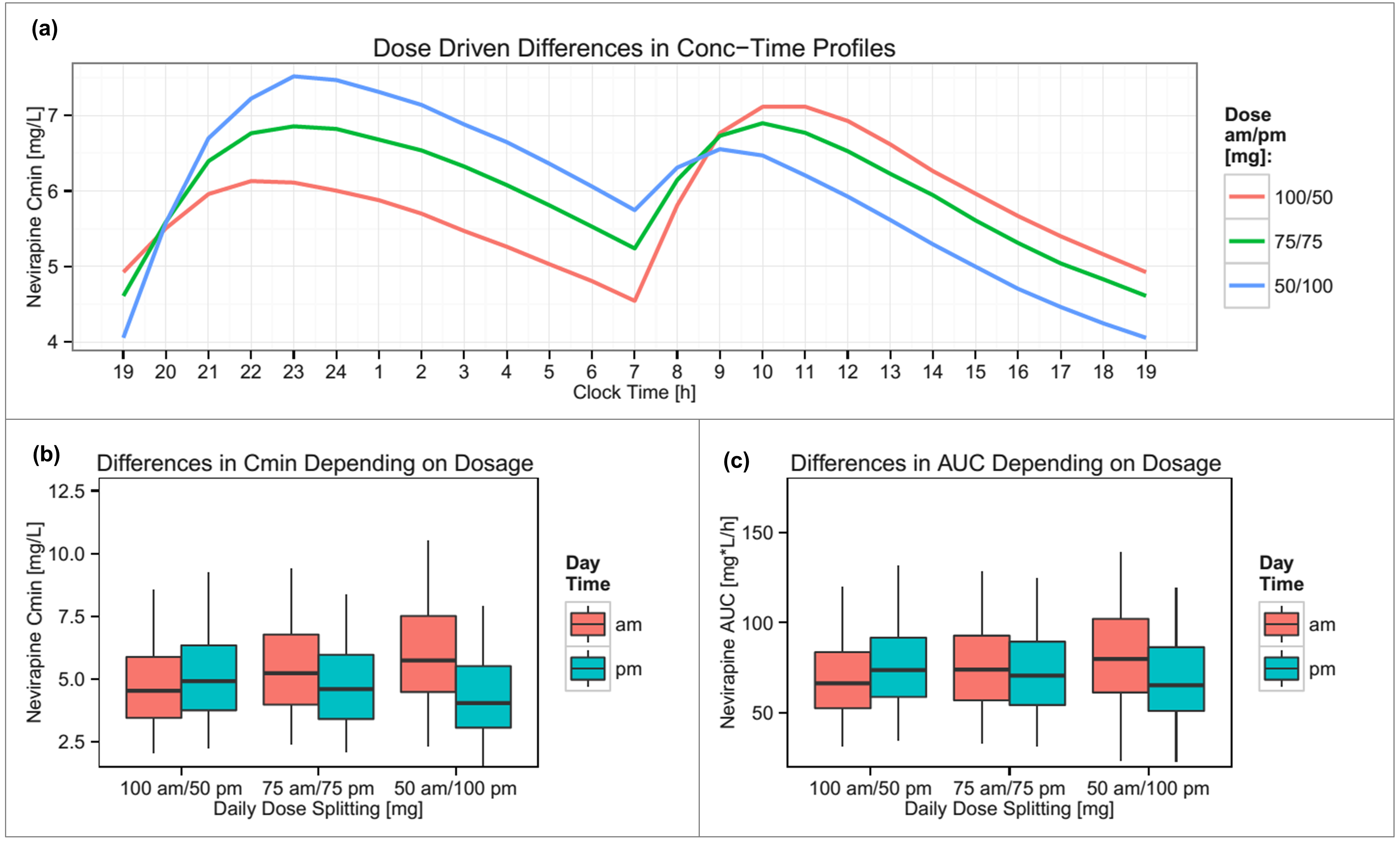


**Figure S3.** Nevirapine exposures obtained using different dose-splitting strategies (see Methods): (a) concentration-time curves for the evaluated dosing scenarios; (b) differences between morning and evening C_min_ depending on dose-splitting strategy; (c) differences between morning and evening AUC depending on dose-splitting strategy.
